# A large cross sectional study on diaper utilization and beneficial role in outdoor activity and emotions among incontinence elderly people

**DOI:** 10.1038/s41598-024-57055-5

**Published:** 2024-03-16

**Authors:** Yunwei Zhang, Dawei Fang, Yashuang Luo, Changying Wang, Lingshan Wan, Yifan Cao, Hongyun Xin, Hansheng Ding

**Affiliations:** 1 Shanghai Health Development Research Center (Shanghai Medical Information Center), Shanghai, 200031 People’s Republic of China; 2 Department of Cardiology, Quanzhou First Hospital Affiliated to Fujian Medical University, Quanzhou, Fujian People’s Republic of China

**Keywords:** Health care, Psychology and behaviour

## Abstract

This study was designed based on a cross-sectional investigation conducted Shanghai, China. Demographic characteristics, diaper utilization, Activities of Daily Living (ADL) and emotion were collected by Unified Needs Assessment Form for Elderly Care Questionnaire. Cognition function was assessed by Mini-mental State Examination (MMSE) scale. Multivariate logistic regression was used for statistical analysis. The diaper utilization rate was 31.2%. Female, higher level of education, poorer ADL and cognition, more severe incontinence and financial dependence on others were facilitating factors for diaper usage (*P* < 0.05). The possibility of using diaper differed according to the intimacy of caregivers. Among incontinent individuals with relatively good ADL and cognition level, diaper utilization can significantly decrease the risk of going out only once a month (OR: 2.63 vs 4.05), and going out less than once a month (OR: 5.32 vs 6.53). Incontinence people who going out at least once a week had a lower risk of some negative emotion. Significantly, diaper utilization further decreased this risk. In conclusion, for incontinence elderly people with relatively independent ability, proper use of diaper may improve the frequency of outdoor activity and emotion. Nevertheless, diaper utilization should be decided based on elderly people’s own will.

## Introduction

Incontinence is a common problem among the elderly people, particularly those of advanced age. The incidence of incontinence in the elderly population ranges from 25 to 45%^1,2^. Incontinence poses a spectrum of adverse effects on the daily and social lives of the elderly, thereby diminishing their quality of life^3^. The decline in the ability to engage in social activity due to embarrassment may lead to social isolation^4^. A research suggested a connection between incontinence and an increased risk of loneliness, anxiety, depression, and poor sleep quality^5,6^. In addition, incontinence contributes to changes in the physical composition and muscle strength degradation in the elderly^7^. Furthermore, it impairs mobility, leading to issues such as inconvenience in movement, falls, and the onset of urinary system infections^8^.

Diapers are commonly employed tools in the care of elderly individuals with incontinence. Elderly individuals can use it at home, during outings, or in social settings, to avoid the awkwardness caused by incontinence, and maintain the privacy^9^. This, in turn, alleviates the caregiver’s workload, enhancing overall care efficiency^10^. Additionally, researches revealed that diaper utilization can facilitate better participation in daily social activity, outings, and other societal and familial engagements, thereby improving the quality of life for the elderly^11^.

The present study was based on a large-scale survey of elderly population, and explored the utilization, effect factors, and potential positive effects of diaper during the outdoor activity and emotion among elderly individuals with incontinence. It aims to provide new evidence and recommendations for the care of elderly individuals with incontinence.

## Methods

### Study population

This study was designed based on a cross-sectional investigation conducted Shanghai, China from January to May 2023. The inclusion criteria was: (1) older adults aged 60 and above; (2) applying for long-term care insurance in Shanghai and voluntarily participating in this study; (3) responded to ‘Do you experience of continence’ and ‘Do you use diaper’ during study. A total of 179,141 older adults were included in the present study.

This study was carried out in accordance with the guidelines proposed in the Declaration of Helsinki, and the study protocol was approved by the Ethics Committee of the Shanghai Health Development Research Center (Approval Number 2020001). All participants signed the written informed consent form prior to commencing the study, and all methods were performed in accordance with relevant guidelines. To ensure the quality of collected data, a training program was given to all investigators before conducting the survey.

### Procedure and data collection

This study conducted by two uniformly trained assessors at the elderly’s home, including a family doctor well-acquainted with the elderly person’s circumstances. The two assessors independently evaluated the daily living and health status of the elderly individuals. Subsequently, the data underwent verification by professionals before being uploaded.

### Incontinence condition and diaper utilization

Incontinence condition and the diaper utilization was conducted by family doctors, then responded to ‘Do you experience of urinary continence’, ‘Do you experience of fecal continence’ and ‘Do you use diaper’. Diaper utilization was categorized into two groups: yes or no. Urinary/fecal incontinence was classified by three groups: with no incontinent, with occasional incontinent, and with incontinent.

### Demographic characteristics

Unified Needs Assessment Form for Elderly Care Questionnaire was used to collect the demographic characteristics of eligible participants. Age, gender, education, main sources of income, living condition, frequency of outdoor activity and number of chronic diseases were collected by investigators based on self-reported or by caregivers. Living condition composed of the following five statuses, ‘Living alone’, ‘Living with spouse’, ‘Living with children’, ‘Living with grandchildren’ and ‘Living with caregivers’. Frequency of outdoor activity composed of the following five statuses, ‘Many times a day’, ‘About once a day’, ‘About once a week’, ‘About once a month’ and ‘Less than once a month’. When analyzed the relationship between outdoor activity and emotions (Table 4), to avoid too few people in some subgroups, elderly people going out many times a day, about once a day and about once a week were merge into group ‘at least once a week’, and elderly people going out about once a month and less than once a month was merge into group ‘once a month or less’. Disease assessment covered 11 chronic diseases, including chronic obstructive pulmonary disease (COPD), diabetes, chronic pneumonia, lower limb fracture, Parkinson’s disease, Ich cerebral hemorrhage (ICH), hypertension, advanced tumor, cerebral infarction, coronary atherosclerotic heart disease and Alzheimer’s Disease. The evaluation of all chronic diseases was carried out based on medical records spanning the most recent six months, with evaluations performed by family physicians who possessed an intimate knowledge of the elderly participants’ medical conditions.

### Assessment of ADL, cognition and emotion characteristics

Cognition level was assessed via Mini-mental State Examination (MMSE) scale, consisting of 30 points in total. ADL was assessed via a self-design scale consisting of 20 items and 20 points in total, this scale was referred from the Activity of Daily Living Scale designed by Lawton and Brody^12^. Emotion characteristics included the following two aspects, ‘Do you feel that you are useless and not being needed?’ and ‘Do you feel more irritable and agitated than usual?’, and were self-assessed by participants.

### Statistical analysis

Univariate analysis was used to explore the associations between demographic and diaper utilization, and variables with statistical significance in univariate analysis were then included in the binary logistic regression model for analysis of effect factors. Multiple logistic regression was used to explore the associations between diaper utilization and outdoor activity. To avoid the confounding effect of functional impairment and cognitive decline, the model included individuals with ADL score above 12.1 points and MMSE score above 18 points. *P* < 0.05 was considered statistically significant. All data were analyzed by using SPSS 23.0 software.

## Results

### Demographic characteristics of participants

The study included a total of 179,141 elderly individuals, with 108,225 in incontinence group and 70,916 in the normal group. Age, ADL, cognition, education, primary economic sources, living status, and health conditions were significantly different between the two groups. The incontinence group comprised individuals who were older, had poorer ADL and cognition, lower levels of education, relied more on external financial support, had a lower proportion of living alone or with family, had a higher proportion of living with caregivers, exhibited lower frequency of outings, and had a higher prevalence of multiple (≥ 3) diseases (Table 1). Besides, a total of 33,716 individuals in the incontinence group used diaper, accounting for 31.2%.

**Table 1:** Demographic characteristics of participants.

Characteristics	Incontinence group (N = 108,225)	Normal group (N = 70,916)	*χ*^2^/*t* value*	*P*
Age (year)	82.26 ± 8.58	78.49 ± 8.58	− 90.88	< 0.001
ADL	10.40 ± 4.56	13.68 ± 3.73	166.82	< 0.001
Cognition	12.12 ± 8.30	17.42 ± 7.82	136.74	< 0.001
Gender (female)	65,414 (60.4)	43,155 (60.9)	3.03	0.08
Education			623.78	< 0.001
Illiteracy	33,137 (30.6)	21,439 (30.2)		
≤ 6 years	22,611 (20.9)	13,415 (18.9)		
7–12 years	35,838 (33.1)	28,637 (40.4)		
> 12 years	8677 (8.0)	5514 (7.8)		
Main sources of income			64.69	< 0.001
Retirement pension	95,759 (88.5)	66,419 (93.7)		
Support from others	4383 (4.0)	2538 (3.6)		
Living alone (Yes)	9038 (8.4)	9906 (14.0)	1743.66	< 0.001
Living with spouse (Yes)	41,536 (38.4)	34,856 (49.2)	2032.44	< 0.001
Living with children (Yes)	42,955 (39.7)	27,889 (39.3)	2.37	0.12
Living with grandchildren (Yes)	2186 (2.0)	1589 (2.2)	10.13	0.001
Living with caregiver (Yes)	21,444 (19.8)	6151 (8.7)	4080.49	< 0.001
Frequency of outdoor activity			11,737.22	< 0.001
Many times a day	230 (0.2)	911 (1.3)		
About once a day	745 (0.7)	2241 (3.2)		
About once a week	4986 (4.6)	8747 (12.3)		
About once a month	20,306 (18.8)	21,761 (30.7)		
Less than once a month	81,958 (75.7)	37,256 (52.5)		
Number of disease			850.10	< 0.001
0	20,051 (18.5)	13,001 (18.3)		
1	36,440 (33.7)	24,127 (34.0)		
2	25,765 (23.8)	19,650 (27.7)		
≥ 3	25,969 (24.0)	14,138 (20.0)		
Diaper use (Yes)			–	–
Urinary incontinence only	8025 (18.2)	–		
Fecal incontinence only	475 (17.6)	–		
Combined incontinence	25,216 (41.1)	–		

*The *t* test was used to evaluated the continuous variables, and the *χ*^2^ test was used to evaluated the categorical variable.

### Effect factors of diaper utilization

Logistic regression analysis was used to explore the effect factors of the diaper utilization. Results showed that women, those with higher educational levels, with poorer ADL and cognitive levels, and experiencing more severe incontinence were more likely to use diaper (*P* < 0.05). Interestingly, the intimacy of caregivers was a significantly effect factor, and diaper may be most likely used among those living with spouse (OR: 0.90), followed by those living with children (OR: 0.88), then grandchildren (OR: 0.69), and was least likely used among those living with caregivers (OR: 0.57) (Table 2).

**Table 2:** Effect factors of diaper use in participants with incontinence.

Characteristics	OR	95% CI	P
Gender (male)	0.84	0.81–0.87	< 0.001
Age	1.01	1.00–1.01	< 0.001
Education			
Illiteracy	1.00	–	–
≤ 6 years	1.86	1.78–1.94	< 0.001
7–12 years	2.18	2.08–2.28	< 0.001
> 12 years	2.13	2.00–2.28	< 0.001
Main sources of income			
Support from others	1.00	–	–
Retirement pension	0.53	0.49–0.57	< 0.001
Living alone (Yes)	1.06	0.98–1.15	0.15
Living with spouse (Yes)	0.90	0.86–0.94	< 0.001
Living with children (Yes)	0.88	0.84–0.93	< 0.001
Living with grandchildren (Yes)	0.69	0.62–0.76	< 0.001
Living with caregiver (Yes)	0.57	0.54–0.60	< 0.001
ADL	0.80	0.79–0.80	< 0.001
Cognition	0.97	0.97–0.98	< 0.001
Urinary incontinence			
Never	1.00	–	–
Sometimes	1.37	1.23–1.54	< 0.001
Always	2.72	2.42–3.07	< 0.001
Fecal incontinence			
Never	1.00	–	–
Sometimes	1.54	1.48–1.59	< 0.001
Always	1.10	1.03–1.17	0.01
Number of disease			
0	1.00	–	–
1	1.08	1.02–1.14	0.01
2	0.93	0.88–0.98	0.01
≥ 3	1.06	1.01–1.12	0.02

Characteristics with statistical significance in previous univariate analyses was involved.

### Role of diaper on outdoor activity in incontinence participants

Logistic regression analysis was used to evaluated the impact of the diaper utilization on the outdoor activity of participants with incontinence. Results indicated that the risk of outdoor activity limitation escalates among incontinent individuals. Compared with going out many times a day, there were about double risk of going out only once a day, and about three times risk of going out only once a week, and about four times risk of going out only once a month, and about six times risk of going out less than once a month. Specifically, among incontinent individuals using diaper, the risk of going out once a month is lower than that of non-users (OR: 2.63 vs 4.05, *P* < 0.05), and the risk of going out less than once a month is also lower than that of non-users (OR: 5.32 vs 6.53, *P* < 0.05, Table 3).

**Table 3:** Relationship between diaper use and frequency of outdoor activity.

Diaper utilization	Frequency of outdoor activity				
	Many times a day	About once a day	About once a week	About once a month	Less than once a month
With incontinence and diaper	1.00	0.62 (0.23, 1.70)	1.95 (0.85, 4.47)	**2.63 (1.16, 5.97)**	**5.32 (2.36, 12.01)**
With incontinence but no diaper	1.00	**1.82 (1.34, 2.48)**	**2.80 (2.10, 3.72)**	**4.05 (3.06, 5.37)**	**6.53 (4.93, 8,64)**

Age, gender, education, living status and number of chronic diseases were included in the model as covariates.

Significant values are in bold.

### Role of diaper on the correlation between outgoing activity and emotion

Outdoor activity may have impact on emotion. Thus an in-depth analysis was conducted to evaluate the correlation between outgoing activity and emotions among incontinent elderly individuals. Compared with elderly people who going out once a month or less, people who going out at least once a week had a lower risk of feeling useless and not being needed (OR < 1, *P* < 0.05). Significantly, diaper utilization further decreased this risk (OR: 0.30 vs 0.77, and 0.11 vs 0.52, *P* < 0.05, Table 4). Among incontinence individuals who not used diaper, outdoor activity may increase the risk of feeling more irritable and agitated than usual (OR: 1.31, *P* < 0.05), but this effect was not found among diaper users (*P* > 0.05).

**Table 4:** Relationship between outdoor activity and emotions.

Diaper utilization	Feel useless and not being needed		Feel more irritable and agitated than usual	
	Often	Sometimes	Often	Sometimes
Yes	**0.30 (0.18, 0.51)**	**0.11 (0.03, 0.39)**	1.32 (0.89, 1.96)	1.15 (0.88, 1.50)
No	**0.77 (0.65, 0.93)**	**0.52 (0.38, 0.71)**	1.04 (0.86, 1.25)	**1.31 (1.20, 1.42)**

The reference group is going out once a month or less, and age, gender, education, living status and number diseases were included in the model as covariates.

Significant values are in bold.

## Discussion

This study was based on a large-scale cross-sectional analysis, revealed a 31.2% utilization rate of diaper among elderly with incontinence. Female, higher level of education, poorer ADL and cognition, more severe incontinence, and financial dependence on others were facilitating factors for diaper usage. Elderly who living with spouses presented the highest likelihood of using diaper, followed by those living with children and then grandchildren, with the lowest likelihood among those living with caregivers. Among elderly individuals with relatively good ADL and cognition, experience of incontinence significantly increased the risk of restricted outdoor activity, which could be mitigated by the use of diaper. Furthermore, outdoor activity can decrease the risk of negative emotion, and diaper utilization made this effect more significant.

The present study have some obvious strengths. Firstly, it provided evidence from large sample real-world. Secondly, the research not only focus the role of diapers in care, but also innovatively indicate the effect of diapers on the physical and mental health of the elderly. Thirdly, a new care strategy may should be carefully considered, that caregivers should neither simply limit or overly apply diaper, and diaper utilization for improving the quality of life for the elderly should be supported. It is important in clinical practice to respect each participant's decision to choose to use diapers or not, if it is based on their own will. A study conducted in Japanese nursing homes revealed a 56% utilization rate of diaper among elderly individuals, with 23.9% was preventive use, slightly higher than the present study. This may be attributed to insufficient staffing in the nursing homes, resulting in an increased reliance on diaper by the elderly due to a lack of assistance with toileting. Additionally, the utilization of diaper is influenced by physical activity levels, daily energy intake, and water consumption^13^. Economic burden and dermatitis^14^ may be important reason why elderly people do not use diapers. A research on economic burden of long-term treatment of severe fecal incontinence revealed that the mean cumulative cost in 10 years per patient on symptomatic treatment was €10,972.9, and diaper expenses account for 62%^15^. Grzybowska et al. reported that 57.3% incontinence women used diaper only during the day, and one-third of women with continence used diapers for 24 h, whose Quality of Life (QoL) and Quality-Adjusted Life Year (QALY) were found lower compared to women using continence pads only during the day^16^.

Some studies revealed that traditional care model tends to excessive restrictions and enforced bedrest^17^. Elderly individuals experiencing urinary incontinence may face restricted mobility, leading to social and psychological issues as well as a decline in functional capacity^18,19^. These observations were similar with the present study, and evidence suggested that continence assessment is helpful to know the volume and frequency of incontinence, then guide the choice of diaper^20^. However, the role of diaper is controversial. Xu et al. found incorrect use of diaper, such as for facilitate care, may contribute to a reduction in the quality of life and personal dignity of the elderly^21^. In the present study, diaper utilization can improve the frequency of outdoor activity and emotion among relatively independent elderly people. Thus, diaper use or not may need to be considered based on specific situations, and if it is for self-supporting care, such as promote going out and social interaction, diaper should be used appropriately. Meantime, the present studies also found that diaper utilization was significantly impacted by intimacy of caregivers, indicated that caregivers should be mindful of respecting the privacy of elderly individuals, offering personalized and comprehensive care strategies, based on each participant's own will^22^.

Outdoor activity is very important for elderly people with incontinence. Firstly, some studies have indicated that physical activity can relieve incontinence^23^, moderate physical activity improving pelvic floor strength and modifying neurophysiological mediators (such as stress) involved in the pathogenesis of incontinence^24^. Secondly, incontinence was found significantly associated with ADL or mobility limitations^18^, and study from Northeast Brazil revealed that older women with incontinence presented dizziness/loss of balance during activities of daily living^25^. ADL/IADL disability and balance impairment are important predictive factors for identifying frailty^26^. Thus, outdoor mobility is benefit for rehabilitation for elderly populations^27^. Thirdly, mobility limitation may cause social isolation and other negative emotions among incontinence patients^28^. The present study also found that outdoor activity can improve some negative emotion. Zhang found that incontinence was a significant risk factor for depression, and the effect of incontinence on depression was completely mediated by ADL^29^. Evidence from a meta-analysis also indicated that depression and anxiety were higher in patients with UI than in those without UI, regardless of age^30^.

There were also some limitations of this study. Firstly, population in this study is a cross-sectional study, which may affect the strength of causal evidence. However, the sample size of this study is very large, which makes a better reliability of the results, and can be better extended to the entire target population. Secondly, the sample population for this study is the elderly who have applied for long-term care insurance, whose health condition may worse than the general population. At the same time, this population included more elderly people with incontinence, and provided more information. Thirdly, in order to improve the acceptance of diapers among the elderly people, some deeper analysis could be focused on reasons why the non-diapers participants refused to use diaper, such as economic status, preferences, etc. Then provide suggestions for diaper improvement in the future.

## Data Availability

The data that support the findings of this study are available on request from the corresponding author. The data are not publicly available due to privacy or ethical restrictions.
